# Nicorandil-induced penile ulceration: a case report

**DOI:** 10.1186/s13256-016-0987-3

**Published:** 2016-07-16

**Authors:** Darren Yap, Omar Aboumarzouk, Christopher Bates

**Affiliations:** Department of Urology, Royal Gwent Hospital, Cardiff Road, Newport, NP20 2UB UK

**Keywords:** Penile, Nicorandil, Ulceration, Nicotinamide, Anti-anginal

## Abstract

**Background:**

Penile ulceration in older patients is commonly neoplastic or infective. However, there are rarer causes of these ulcerations that we have to exclude. We present a rare complication of penile ulceration secondary to nicorandil, a nicotinamide ester.

**Case presentation:**

An 86-year-old white British man was referred with a bloody discharge from under his phimotic foreskin and a lump on the shaft of his penis for the past year. He had angina which has been controlled with nicorandil for the past 5 years. A surgical biopsy of the ulcer showed inflammation with no evidence of malignancy. His penile ulceration spontaneously resolved when he stopped his nicorandil treatment.

**Conclusions:**

Nicorandil-induced ulceration is a rare complication; however, it should not be missed in a clinical setting. If there is any doubt about the cause of penile ulceration, then referral to dermatology or urology for consideration of biopsy is essential.

## Background

Penile ulceration in older patients is most commonly neoplastic or infective in nature. A biopsy is usually required to exclude malignancy and subsequent treatment depends on the histopathological results.

An extremely rare cause of penile ulceration is a complication from nicorandil; in this case, nicorandil was part of a treatment for angina. At present there are only six reported cases in the English literature. In this case report, we report the seventh case and review the available literature on this subject.

## Case presentation

An 86-year-old white British man was referred with a bloody discharge from under his phimotic foreskin and a lump on the shaft of his penis. He had noticed the lump following catheterization, during an episode of pancreatitis, 1 year before presentation. He had a past medical history of atrial fibrillation, angina, chronic obstructive pulmonary disease (COPD), and ischemic heart disease. He had an angioplasty 7 years ago and was not suitable for any cardiac surgery. He had been on nicorandil for 5 years which had previously caused him mouth ulcers. His mouth ulcers were previously reviewed by oro-maxillofacial surgeons, and nicorandil was switched to ranolazine on the advice of a cardiologist. Even though his mouth ulcers resolved spontaneously, he responded unfavorably to ranolazine, hence he returned to nicorandil.

**Fig. 1:**
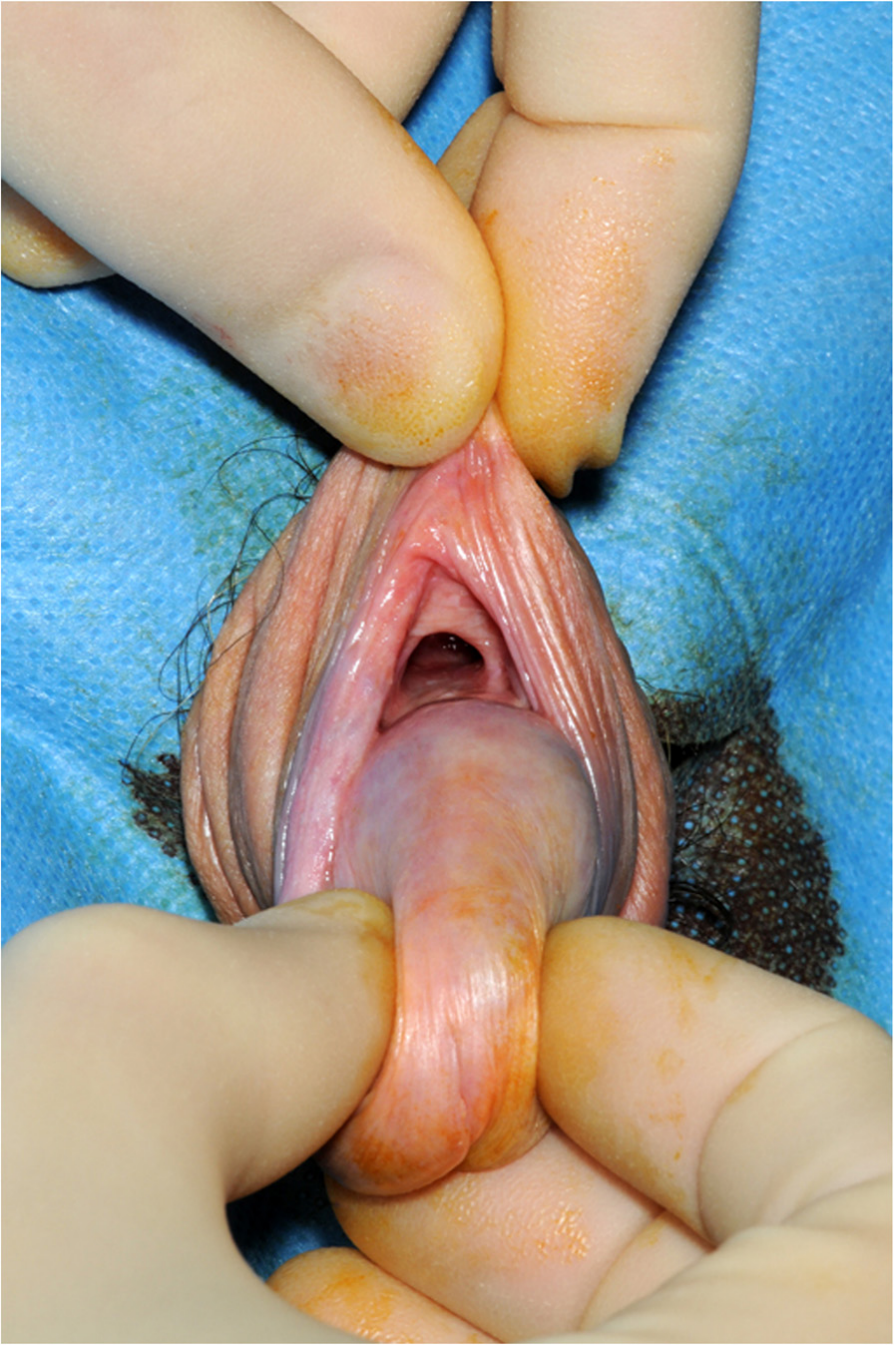
Penile ulceration at dorsal surface

On examination, there was inflammation of his penile shaft skin and foreskin. His foreskin was unable to be retracted fully and there was some serosanguineous discharge on the superior aspect of his foreskin. A cardiovascular examination reveal normal irregularly irregular heart sounds with no evidence of murmur. He was short of breath at rest and on auscultation he had coarse vesicular breath sounds across both lungs. Surgical exploration revealed an inflamed ulcer under his foreskin on the dorsal aspect of his penis extending into deep tissues from the coronal sulcus (Fig. 1). A biopsy was performed which showed inflammation with no evidence of any malignancy.

His nicorandil treatment was stopped and he was started on isosorbide mononitrate (Monomil XL) under the advice of a cardiologist. At follow up, 10 weeks later, there was significant improvement in his penile ulceration. He did not require any further intervention for his ulceration. He also responded well to the isosorbide mononitrate in the treatment of his angina.

## Discussion

Nicorandil is a type of nicotinamide ester used for the treatment of angina and ischemic heart disease [1–3]. It has a dual mechanism of action by firstly donating a nitric oxide to activate guanylate cyclase and secondly activating Adenosine triphosphate (ATP)-sensitive potassium channel [1, 3]. The vasodilatory effect of nicorandil reduces the cardiac preload and afterload increasing coronary blood flow [1, 2].

A large, prospective randomized controlled trial named Impact of Nicorandil in Angina (IONA) has demonstrated the efficacy of nicorandil in reducing cardiovascular mortality and morbidity for patients receiving nicorandil in comparison to those on a placebo [4]. Common side effects of this medication include nausea, vomiting, rectal bleeding, flushing, tachycardia, dizziness, headache, and weakness [5]. Less commonly it can cause ulcers in the oral mucosa and in anal, perianal, and parastomal cutaneous sites [1–3, 5]. Recently there have also been several reports of penile ulceration in the literature [1–3, 6, 7].

There is a wide range of differential diagnoses for penile ulceration which can be divided into three main categories: malignancy, non-infectious, and infectious as summarized in Table 1 [1, 3, 8]. Infectious causes include syphilis, chancroid, herpes simplex virus, granuloma inguinale, and lymphogranuloma venereum [1, 3, 8]. Crohn’s disease, aphthous ulcers, Behçet’s disease, factitial dermatitis, Wegener’s granulomatosis, leukocytoclastic vasculitis, and pyoderma gangrenosum are examples of non-infective or inflammatory causes [1, 3, 8]. Lastly, malignant causes are basal cell carcinoma, squamous cell carcinoma, and sarcoma [1, 3, 8].

**Table 1:** Differential diagnosis [1, 3, 8]

Infectious	
• Syphilis	
• Chancroid	
• Herpes simplex	
• Granuloma inguinale	
• Lymphogranuloma venereum	
Non-infectious	
• Crohn’s disease	
• Aphthous ulcer	
• Behçet’s disease	
• Factitial dermatitis	
• Wegener’s granulomatosis	
• Leukocytoclastic vasculitis	
• Pyoderma gangrenosum	
Malignancy	
• Squamous cell carcinoma	
• Basal cell carcinoma	
• Sarcoma	

Nicorandil-induced penile ulceration is a diagnosis of exclusion; a biopsy may be necessary to exclude malignancy and in sexually active individuals it is important to exclude sexually transmitted infections such as syphilis which can present with similar lesions [1, 3]. Other than a tissue diagnosis for histology, blood tests should be performed to exclude vasculitic causes of the penile ulceration.

Nicorandil-induced ulcers tend to be well demarcated and deep with histology showing acute inflammation [1]. The pathogenesis of this rare side effect is still not clearly understood. Direct toxicity where there is abnormal accumulation of nicorandil or one of its metabolites and localized hypersensitivity reaction has been postulated as a possible cause [1, 8]. The second hypothesis is known as the “vascular steal” effect which suggests that nicorandil causes redistribution of arterial and venous flow having a profound effect on penile end arteries [1, 8].

The onset of penile ulceration is similar to reported oral and anal ulceration which varies from several weeks to months after starting on nicorandil [2, 8]. The effects of these ulcers are reversible on stopping the medication (nicorandil) [1]. However, if the ulcers are particularly large, then the defect may not heal and surgical intervention might be required [1]. Resolution of the ulcer may take up to 12 weeks after drug withdrawal [8].

Penile ulceration can be clinically challenging to treat and manage. It is highly recommended to arrange an urgent review by dermatology or urology to aid differentiating the ulcer from other malignant causes [7]. It is also crucial to discuss with a cardiologist the consideration of an alternative medical therapy as the cessation of nicorandil may result in serious complications [7].

## Conclusions

We present a case of nicorandil-induced penile ulceration which resulted in complete resolution after surgical exploration and cessation of the medication. If there is any doubt about the cause of penile ulceration, then referral to dermatology or urology for consideration of biopsy is essential. Alternative medical therapy is important, as sudden cessation of nicorandil may result in serious cardiac consequences in some patients [7]. Cessation of the medication can lead to resolution of the ulcer avoiding unnecessary and potentially harmful surgery [8].

## Patient’s perspective

I first came into contact with nicorandil’s side effects when I began to get mouth ulcers. My dentist sent me to the Royal Gwent Hospital (RGH) Dental department and after tests they found nicorandil was the culprit. This was cured with Corsodyl (the active ingredient of Corsodyl mouthwash is chlorhexidine digluconate). Sometime later my foreskin became enlarged and inflamed. I was prescribed various creams to no avail. I was referred to Dr Bates who said he had only seen this once before, but it was due to medication. I mentioned the mouth ulcers and he said that was it nicorandil. The answer was surgery, which he performed after taking photographs. I have stopped nicorandil and to date have had no further trouble.
